# Pseudo Broad Ligament Fibroid Posing a Clinical Dilemma: A Case Report

**DOI:** 10.7759/cureus.43272

**Published:** 2023-08-10

**Authors:** Megha Karnik, Neema Acharya, Jyotsna Potdar, Shaikh Muneeba, Preeti Mishra, Samarth Shukla

**Affiliations:** 1 Obstetrics and Gynecology, Jawaharlal Nehru Medical College, Datta Meghe Institute of Higher Education and Research, Wardha, IND; 2 Pathology, Jawaharlal Nehru Medical College, Datta Meghe Institute of Higher Education and Research, Wardha, IND

**Keywords:** degeneration, leiomyomas, hysterectomy, broad ligament, ovarian cyst

## Abstract

Pseudo broad ligament fibroids originate in the uterus but grow into the broad ligament while retaining their attachment to the uterus. We report a case of a right-sided pseudo broad ligament fibroid mimicking an adnexal mass in a 40-year-old woman presenting with dysmenorrhoea and heavy menstrual bleeding. She had a uterine mass corresponding to an 18-week pregnant uterus size, which clinically and radiologically posed a diagnostic dilemma between an ovarian cyst, a bicornuate uterus with multiple fibroids, and a broad ligament fibroid. She underwent a total abdominal hysterectomy with bilateral salpingo-oophorectomy, which revealed a pseudo broad ligament fibroid of 12x10x6 centimeters, weighing 1.2 kg. The histopathology report confirmed the diagnosis of a leiomyoma with normal ovaries.

## Introduction

Fibroids in the broad ligament, though rare, occurring in less than 1% of all fibroids, are the most common extrauterine leiomyomas [1]. Their varied appearance may make a preoperative diagnosis difficult. We report here a case of a large false broad ligament fibroid causing a diagnostic dilemma between a uterine anomaly and an ovarian tumor.

## Case presentation

A 40-year-old female, para one, with a previous cesarean section, non-tubectomized, presented with complaints of heavy menstrual bleeding and dysmenorrhoea for one year. There were neither disturbances in her bowel and bladder habits nor a history of weight loss. There were no associated comorbidities and no history of ovarian or breast cancers in the family. On general examination, she had a BMI of 21 kg/m2 and mild pallor. Her pulse was 88 beats per minute, regular and with good volume, and her blood pressure was 118/80 mmHg in her left arm in the supine position. Her abdominal examination revealed a mass in the hypogastrium corresponding to an 18-week-old uterus. It was palpable more on the left side within the hypogastric region. However, its lower margin was not palpable. Another mass corresponding to an 18-week-old uterus was palpated on the right, adjacent to the above mass. This mass was firm and regular, with a positive groove sign (a groove present between the two masses, indicating probable adnexal origin). The lower extent of the mass was difficult to ascertain. On per-speculum examination, a single cervix was seen. The cervix and vagina were unremarkable. Per vaginal examination, an anteverted, 18-week-size uterus was revealed that was separate from the mass. The mass was smooth and firm and occupied the posterior fornix. Bilateral fornices were full but non-tender. We suspected a fibroid or an adnexal mass.

**Figure 1:**
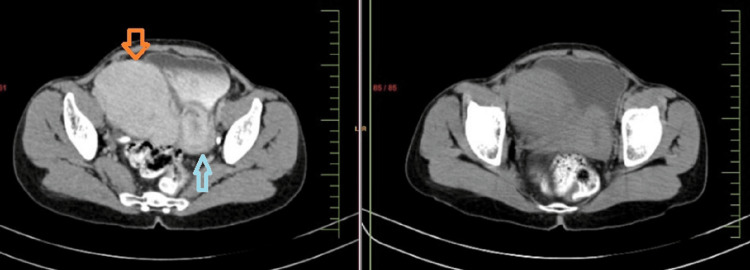
A computerized tomography image of the pelvis shows a pseudo broad ligament fibroid (orange arrow) and its lateral relation to the uterus (blue arrow).

Her routine investigations were within normal limits. Her cancer antigen (CA) 125 levels were 6.8 U/ml (normal range: 0-35 U/ml). Preliminary ultrasonography of the abdomen revealed a suspected bicornuate uterus with a bulky right horn. However, the opinion of a senior radiologist after repeating detailed ultrasonography revealed a bulky uterus with a submucosal fibroid measuring 3x3 cm and an adnexal mass measuring 10x8 cm, most likely a subserosal or broad ligament fibroid. Computerized tomography (CT) in Figure 1 showed a bulky uterus with a submucosal fibroid of 5x5x3 cm.

A well-defined heterogeneously enhancing soft tissue density lesion was noted in the right adnexa measuring 11x10x10 cm, which was seen separately from the ovary. The mass derived its blood supply from the internal iliac artery and caused the displacement of the pelvic organs. The CT was suggestive of a broad ligament fibroid. The patient was worked up for laparotomy with a total abdominal hysterectomy.

**Figure 2:**
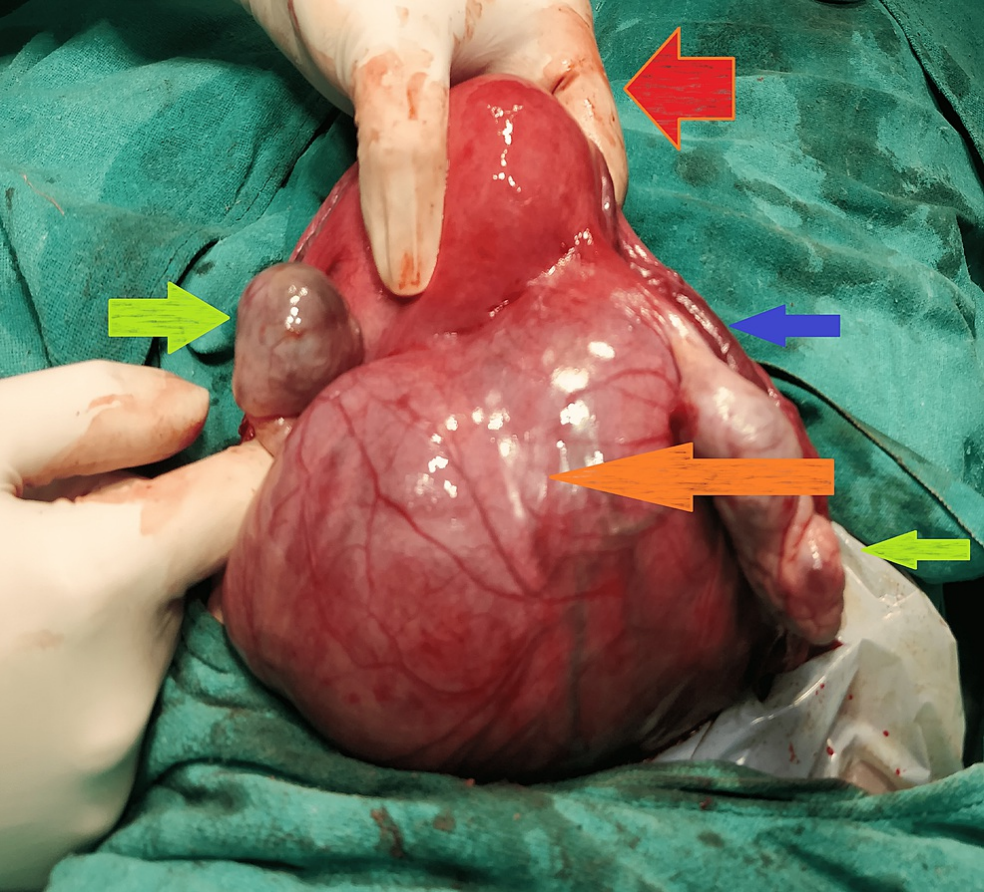
The intraoperative image shows a large, right-sided pseudo broad ligament fibroid (orange arrow) with bilaterally normal ovaries and uterus (green and red arrows, respectively). The right fallopian tube (green arrow) is stretched over the fibroid. The fibroid had to be enucleated to prevent injury to the ureter, which was lying lateral to the fibroid.

The patient underwent a laparotomy under spinal anesthesia. Figure 2 shows evidence of a large, broad ligament fibroid on the right side measuring 12x10x6 cm, which was firm but smooth.

The right fallopian tube was pulled over the fibroid. A loop of the small intestine was adhered to the fibroid by thin adhesion, likely suggesting a parasitic blood supply. The left-sided round ligament was stretched, and the uterus was deviated to the left. The uterus was eight to 10 weeks in size and was unremarkable. The ureteric course was determined to be lateral to the fibroid, suggesting its uterine origin.

**Figure 3:**
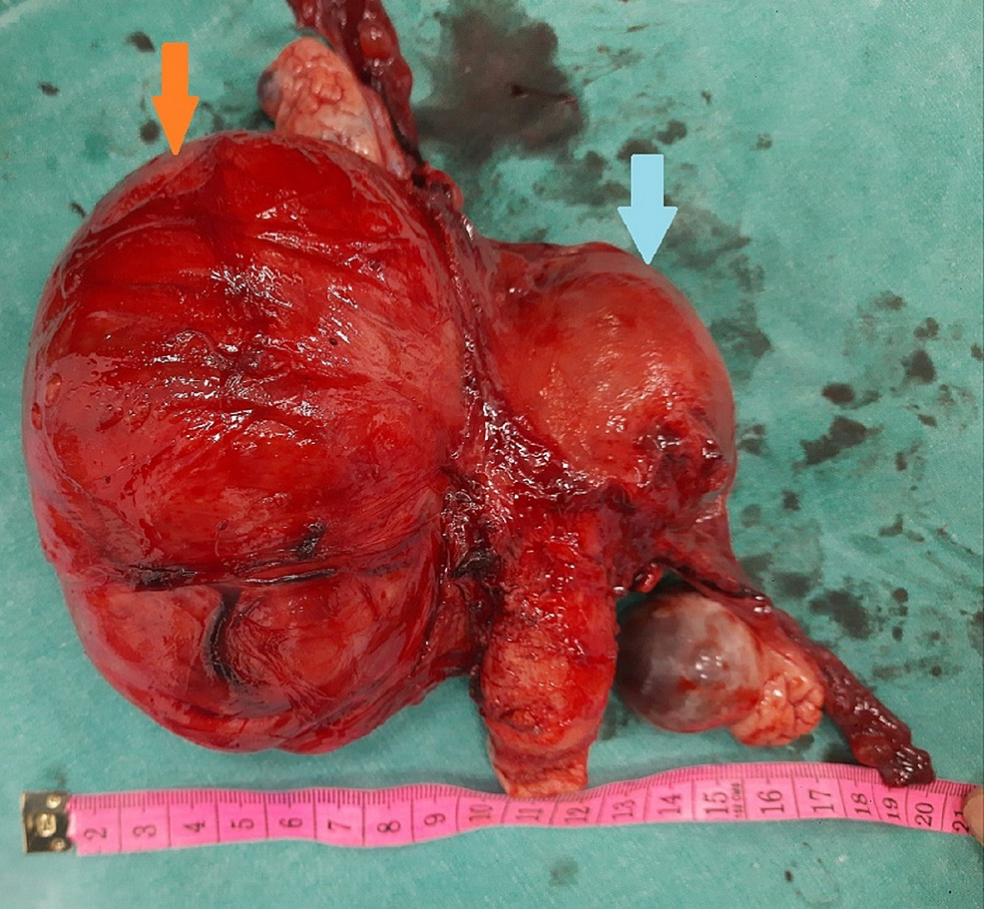
The operated specimen shows attachment of the pseudo broad ligament fibroid to the lateral uterine wall (orange arrow), a bilateral normal adnexa, and an unremarkable uterus with the cervix (blue arrow).

She underwent a total abdominal hysterectomy with bilateral salpingo-oophorectomy by the routine method as seen in Figure 3 after carefully dissecting the fibroid and the bowel.

**Figure 4:**
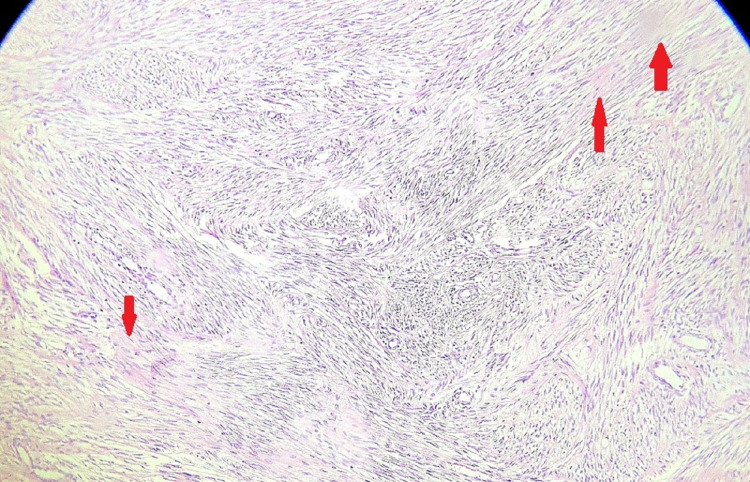
Microscopic appearance Haematoxylin and eosin (H&E) staining at 100x magnification shows intersecting fascicles of spindle cells with cigar-shaped nuclei and a whorled pattern with areas of hyaline degeneration (red arrow) suggestive of leiomyoma undergoing hyaline degeneration.

The patient had an uneventful recovery in the postoperative period. The histopathology report showed a leiomyoma with hyaline degeneration, as seen in Figure 4.

## Discussion

Fibroids, the most common neoplastic growth in the uterus, may occur at extrauterine sites, the broad ligament being the most common site. Broad ligament fibroids (FIGO 8) are either true, i.e., primary, or false, which is secondary. True broad ligament fibroids arise from the smooth muscle tissue in the broad ligament. False broad ligament fibroids originate in the uterus but grow into the broad ligament while retaining their attachment to the uterus. The ureter and uterine artery run laterally to the fibroid, which may coexist with uterine fibroids [2].

A broad ligament fibroid may cause compression symptoms like urinary and bowel dysfunction, depending on the organ being compressed. When coexisting with intramural or submucosal fibroids, they may cause menstrual symptoms [3], as was the case in our case. When present in the broad ligament, these fibroids may pose difficulty in preoperative diagnosis and require higher modalities of radiological investigations [4]. The differential diagnosis in such cases is usually ovarian (benign or malignant) neoplasia, tubo-ovarian masses, broad ligament cysts, and lymphadenopathy [5]. In this patient, clinical findings and ultrasonography raised the suspicion of a neoplastic mass of the ovary or subserosal fibroid or a bicornuate uterus with multiple fibroids. The serum level of CA-125 was within the normal range.

Large, broad ligament leiomyomas often displace adjacent organs and distort pelvic anatomy, increasing the risk of injury to the ureters and adjoining structures [6]. Its location and large size, in our case, made exposure difficult for the surgery. The fibroid was dissected from its pseudocapsule to avoid injury to the ureter, and a pan-hysterectomy was performed. In our case, we also found a few feeding vessels at the base of the pseudo broad ligament leiomyoma, the bridging vessel sign, which helped locate its uterine origin [7]. These vessels were a potential source of excessive bleeding during the surgery. These were coagulated, and adhesions from the bowel were released. When these fibroids grow to large sizes, secondary changes like degeneration, infection, hemorrhage, and necrosis may occur [8, 9]. Hyaline degeneration is the most common degenerative change in fibroids, followed by other degenerations like myxomatous changes, calcification, mucoid changes, cystic degeneration, red degeneration, and very rarely fatty changes [10,11]. Cystic degeneration at times mimics a metastatic ovarian mass, which further complicates the preoperative diagnosis. In our case, despite its huge size, the fibroid had not undergone any cystic degenerative changes. However, areas of hyaline degeneration were present on histopathological examination. Preoperative stenting of the ureter is one of the ways to prevent injury to the ureter [12]. However, due to the financial constraints of the patient, we were unable to do the same, and other surgical precautions were followed to prevent ureteric injury.

## Conclusions

Broad ligament fibroids pose a diagnostic challenge for both the gynecologist and the radiologist. An ultrasound may not suffice to differentiate these from ovarian masses, and other aids like an MRI or CT scan would be required. Concurrent uterine fibroids may be present, causing menstrual symptoms along with pressure symptoms. The surgical approach must be kept in mind to avoid injury to the ureters and surrounding structures due to distorted anatomy. Preoperative ureteric stenting can be considered to avoid injury to the ureters in cases of large and unusual fibroids. Our case highlights the need to keep in mind the various differential diagnoses while dealing with suspected adnexal masses, the diagnostic challenges faced, and the surgical approach required to reduce injuries while operating on broad ligament fibroids.
